# Impact of antiviral treatment on long-term prognosis in non-immunocompromised patients with CMV reactivation

**DOI:** 10.1186/s12879-021-06098-4

**Published:** 2021-05-04

**Authors:** Ga Eun Park, Hyun Kyun Ki, Jae-Hoon Ko

**Affiliations:** 1 Division of Infectious Disease, Konkuk University medical center, Konkuk University School of Medicine, Seoul, Republic of Korea; 2 Division of Infectious Diseases, Department of Medicine, Samsung Medical Center, Sungkyunkwan University School of Medicine, 81 Irwon-ro, Gangnam-gu, Seoul, 06351 Republic of Korea

**Keywords:** Cytomegalovirus, Reactivation, Ganciclovir, Long-term, Prognosis

## Abstract

**Background:**

Reactivation of human cytomegalovirus (CMV) occurs in non-immunocompromised patients with or without specific organ involvement, but it is still unknown whether it has a clinical implication on long-term prognosis or not.

**Methods:**

A retrospective cohort study evaluating non-immunocompromised adult patients with CMV reactivation was conducted during the period between January 2010 and February 2018. Patients were divided into ganciclovir-treated and non-treated groups. Patients who died within 30 days from CMV reactivation were excluded as they died from complex causes of conditions. Survivors were followed for 30-months to evaluate long-term prognosis.

**Results:**

A total of 136 patients with CMV reactivation was included, consisting of 66 ganciclovir-treated (48.5%) and 70 non-treated (51.5%) patients. Overall, patients were old-aged (median 70 years old) and most were treated with pneumonia of any cause (91.2%). More patients in ganciclovir-treated group were treated at intensive care unit (43.9% vs 24.3%, respectively) and had higher viral load over 5000 copies/ml (48.5% vs 22.9%) than non-treated group (all *P* < 0.05). Primary and secondary endpoints including 30-months survival (28.0 vs 38.9%, respectively) and 12-months survival (40.3% vs 49.2%) were not statistically different between the ganciclovir-treated and non-treated groups. In the multivariate analyses, ganciclovir treatment was not associated with 30-months survival (HR 1.307, 95% CI 0.759–2.251) and 12-months survival (HR 1.533, 95% CI 0.895–2.624).

**Conclusion:**

In a retrospective cohort study evaluating non-immunocompromised patients with CMV reactivation, ganciclovir treatment was not associated with long-term prognosis. Antiviral treatment in this condition would not be necessary unless organ involvement is suspected.

**Supplementary Information:**

The online version contains supplementary material available at 10.1186/s12879-021-06098-4.

## Introduction

Human cytomegalovirus (CMV) is an important viral pathogen in immunocompromised patients, especially those with human immunodeficiency virus (HIV) infection, receiving solid organ transplantation (SOT), or receiving hematopoietic stem cell transplantation (HCST) [1, 2]. Non-immunocompromised patients, broadly defining those without HIV infection or exogenous immune-suppression, had not been considered at-risk populations for CMV diseases. However, growing evidence suggests that CMV reactivation also occurs in non-immunocompromised patients with severe illness and would be associated with higher mortality and prolonged hospitalization [3–12]. CMV reactivation in these hosts could be an indicator of severe illness rather than a determinant, but it has also been suggested that CMV infection may cause chronic inflammation and potentially associated with adverse outcomes such as cardiovascular events [13]. It is still unclear whether CMV reactivation would cause chronic inflammation and has adverse effects on long-term prognosis. Because the clinical significance of CMV reactivation is unknown, treatment with patients with CMV reactivation has not been determined and the impact of antiviral treatment for CMV reactivation on long-term clinical outcome in non-immunocompromised patients is also unknown [6, 8, 14]. Ganciclovir, a synthetic analogue of 2′-deoxy-guanosine with antiviral activity against CMV, is usually administered in immunocompromised patients, but we also administered ganciclovir to several critically ill, non-immunocompromised patients with CMV reactivation. We retrospectively compared whether the long-term prognosis differed by antiviral treatment in non-immunocompromised patients with CMV reactivation.

## Methods

### Study desing and population

**Fig. 1:**
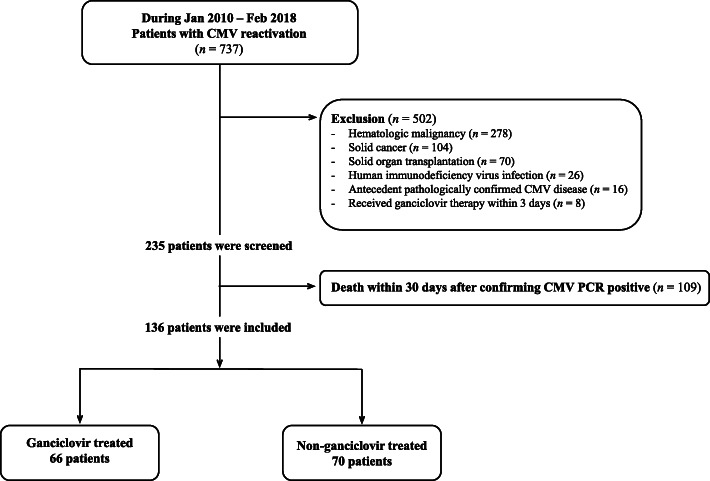
Flowchart of study population among patients with CMV reactivation. Abbreviations: CMV, cytomegalovirus, PCR, polymerase chain reaction

This is a retrospective cohort study evaluating patients with CMV reactivation at Konkuk University Hospital, an 850-bed, community-based tertiary medical center in Seoul, Republic of Korea, during the study period between January 2010 and February 2018. Patients were tested for CMV DNAemia using real time polymerase chain reaction (RT-PCR), based on the clinical judgement of the attending physician suspecting CMV reactivation. CMV reactivation was defined as detection of CMV DNAemia (> 270 copies/ml) in whole blood by RT-PCR. Patients with clinical presentation suggesting primary CMV infection, such as infectious mononucleosis-like illness or fever of unknown origins were not included. Patients with hematologic or oncological disorders, with HIV infection, with previous history of pathologically confirmed CMV disease, those who had received SOT, or those who discontinued ganciclovir within 3 days or received other antiviral agents were excluded. Patients who died within 30 days were also excluded from the analysis (Fig. 1). Patients were divided into ganciclovir treatment group and non-treated group. Ganciclovir was administered at 5 mg per kilogram every 12 h, and the dose was adjusted according to patients’ renal function. The dose was not adjusted according to the amount of CMV PCR. We additionally included a third group of non-immunocompromised patients who tested negative for CMV infection, matching age, sex, and length of hospital stay after CMV test. The present study was approved by the Institutional Review Board (IRB) of Konkuk university of medical center (#2020–04-040). Informed consent was waived by the IRB of Konkuk university of medical center since the electronic medical record (EMR) was reviewed retrospectively with de-personalized identification number.

### Clinical data collection

Data were collected from hospital database including the administrative, pharmaceutical, and laboratory information at Konkuk university Hospital. EMRs were reviewed, including treatment with ganciclovir, age, sex, underlying disease, intensive care unit (ICU) stay, length of hospital stay after CMV reactivation, mechanical ventilation, presence of pneumonia, and maximum titer of CMV PCR. CMV pneumonitis was defined as a case of atypical infiltration on Chest X-rays, progression of X-ray lung lesions despite broad spectrum antibiotic use, and bronchoaveolar lavage (BAL) or sputum CMV PCR of 10,000(copies/ml) or more [15]. Diagnosis of CMV pneumonitis was made regardless of concomitant bacterial infection. The severity of underlying disease at the time of CMV reactivation was estimated using Charlson’s weighted index of comorbidity (CWIs). The severity of illness at the time of CMV reactivation was assessed using quick sequential organ failure assessment (qSOFA) score. The primary endpoint was the 30-months survival. The secondary endpoints were 90-days and one-year survival.

### CMV PCR assays

The Real-Q CMV DNA quantification kit (Real-Q assay; BioSewoom, Seoul, Korea) was used for detection of CMV DNA in whole blood. The kit is designed to detect CMV genome in purified DNA samples via the gene coding for the glycoprotein B (gB). DNA was extracted on a MagNA Pure 96 instrument (Roche Diagnostics, Mannheim, Germany) with the “Pathogen Universal Protocol” (elution volume, 100 μL), according to the manufacturer’s recommendation. Detection and quantification of CMV DNA was performed by using the Real-Q assay. The PCR reaction was performed in a total volume of 25 μL (15 μL of PCR reaction mixture including probe and primers, plus 10 μL of template DNA). Results are indicated in copies per milliliter of whole blood (copies/ml) [16]. Additional tests for ganciclovir-resistant mutations were not conducted.

### Statistical analysis

The baseline characteristics were compared between the ganciclovir-treated and non-treated groups, using Mann-Whitney U test for continuous variables and Fisher’s exact tests for categorical variables. We used Cox proportional hazard regression analysis to evaluate the association between ganciclovir treatment and long-term prognosis. In the multivariate-adjusted model, we included ICU stays, qSOFA score, CWIs, age, CMV PCR titer more than 5000 copies/ml, and variables with statistical significance in the univariate analysis were included in the multivariate analysis. The same statistical analyses were conducted for the comparison between CMV-positive and negative patients. For all analyses, a two-tailed *p* value < 0.05 was considered statistically significant. IBM SPSS Statistics version 25.0 for Windows (IBM, Armonk, NY, USA) was used for all statistical analyses.

## Results

### Patients characteristics

A total of 737 patients showed CMV DNAemia during the study period. After excluding immunocompromised hosts, patients died within 30 days, and patients with other exclusion criteria, 136 adult patients with CMV DNAemia were finally included in the study cohort for the evaluation of long-term prognosis (Fig. 1). Demographic characteristics of the patients between ganciclovir-treated (66 patients, 48.5%) and non-treated group (70 patients, 51.5%) are shown in Table 1. There were no significant differences between the ganciclovir-treated group and non- treated group with regard to age, sex, mechanical ventilation, qSOFA and CWIs. The proportion of patients who stayed in ICU (43.9% vs. 24.3%, respectively, *P* = 0.015) and who had > 30 days of hospitalization (63.6% vs 44.3%, respectively, *P* = 0.024) were higher in the ganciclovir-treated group compared to the non-treated group. The number of patient with CMV PCR titer over 5000 copies/ml is higher in ganciclovir-treated group than non-treated group (48.5% vs 22.9%, respectively, *P* = 0.002).

**Table 1:** Baseline characteristics of patients between ganciclovir-treated and non-treated group

Variables	Ganciclovir treated (*n* = 66)	Non-treated (*n* = 70)	***P*** value
**Sex, male**	47 (71.2)	40 (57.1)	0.088
**Age (years)**	71.5 (62.3–80.0)	69 (59.8–76.0)	0.419
**Pneumonia**	60 (90.9)	64 (91.4)	0.915
**Severity variables**			
ICU	29 (43.9)	17 (24.3)	0.015
Mechanical ventilation	16 (24.2)	10 (14.3)	0.140
Quick SOFA	1 (0.0–2.0)	1 (1.0–1.0)	0.650
CWI	1 (1.0–2.0)	1 (0.0–1.3)	0.177
HD > 30 days after CMV reactivation	42 (63.6)	31 (44.3)	0.024
CMV PCR > 5000 copies/ml	32(48.5)	16 (22.9)	< 0.002
CMV DNAemia duration (days)	12 (9.0–19.0)	8.5 (7.0–15.5)	0.137
Progress to CMV pneumonitis	10 (15.2)	0 (0.0)	0.001
**Endpoint**			
30-months survival	14/50 (28)	21/54 (38.9)	0.240
One-year survival	25/62 (40.3)	30/61 (49.2)	0.323
90-days survival	47/63 (74.6)	60/69 (87)	0.070
Cardiovascular events	4 (6.1)	2 (2.9)	0.363

Data are expressed as number (%) of patients or median (IQR)

*Abbreviations: ICU* intensive care unit, *SOFA* sequential organ failure assessment, *CWI* Charlson’s weighted index of comorbidity, *HD* hospital day *CMV* Cytomegalovirus, *PCR* polymerase chain reaction

### Long-term prognosis in patients with CMV reactivation

In the univariate analysis, no variables were positively associated with 30-months survival (Table 2). In the multivariable analysis, qSOFA score (HR 1.472, 95% CI 1.001–2.163, *P* = 0.049) were significantly associated with 30-months survival. Ganciclovir treatment was not associated with 30-months survival (HR 1.307, 95% CI 0.759–2.251, *P* = 0.334). The one-year survival also showed statistically similar results. In the multivariable analysis, qSOFA score was significantly associated with one-year survival (HR 1.595, 95% CI 1.049–2.426, *P* = 0.029). Ganciclovir treatment was not associated with 90-days and one-year survivals (Table S1 and S2). Ten out of 66 patients (15.2%) who treated with ganciclovir probably progressed to CMV pneumonitis, which showed BAL or sputum CMV PCR over 10,000 copies/ml. On the other hand, no patient out of 70 patients who were not received ganciclovir progressed to CMV pneumonitis. Four patients out of 136 patients developed cardiovascular event, but there were no statistically differences between ganciclovir-treated group and non- treated group. (6.1% vs 2.9%, respectively, *P* = 0.363). We further analyzed the 30-months survival between patients who undergone CMV test but were negative and patients with CMV reactivation. One hundred thirty-one patients were further analyzed by matching the CMV activation group (Table S3). Regardless of whether ganciclovir was treated or not, CMV reactivation did not affect the 30-month survival rate in the multivariate analysis (Table S4).

**Table 2:** Association between baseline characteristics of patients and 30-months survival

Variables	Univariate		Multivariate	
	HR (95% CI)	*P* value	HR (95% CI)	*P* value
Sex	1.231 (0.739–2.050)	0.425		
Age	0.985 (0.970–1.001)	0.062	0.987 (0.971–1.004)	0.132
Ganciclovir treatment	1.175 (0.727–1.898)	0.511	1.307 (0.759–2.251)	0.334
Pneumonia	1.353 (0.543–3.369)	0.516		
ICU	0.943 (0.566–1.570)	0.821	0.656 (0.354–1.213)	0.179
Mechanical ventilation	1.367 (0.779–2.400)	0.276		
Quick SOFA	1.326 (0.935–1.880)	0.113	1.472 (1.001–2.163)	0.049
CWI	0.884 (0.689–1.135)	0.335	0.904 (0.699–1.168)	0.796
Hospital days > 30 days	0.893 (0.553–1.442)	0.644		
CMV PCR > 50,000 copies/ml	1.094 (0.648–1.848)	0.736	0.926 (0.519–1.654)	0.796
Progress to CMV disease	0.377 (0.092–1.541)	0.175		
Cardiovascular event	1.276 (0.312–5.221)	0.734		

*Abbreviations: ICU* intensive care unit, *SOFA* sequential organ failure assessment, *CWI* Charlson’s weighted index of comorbidity, *CMV* Cytomegalovirus, *PCR* polymerase chain reaction

## Discussion

There was no statistically significant association between ganciclovir treatment and long-term prognosis in the multivariate analysis. As far as we know, this is the first study to compare long-term prognosis between ganciclovir-treated and non- treated group in non-immunocompromised patients with CMV reactivation. There are several studies on the association between CMV reactivation and poor prognosis such as mortality, longer hospitalization and mechanical ventilation among critically-ill immunocompetent patients [5–7, 11, 17–19]. Although the direct adverse effects of CMV infection on various organs have been suggested [11, 20], clinical significance of CMV reactivation based solely on blood CMV titers is still unknown. In addition, whether CMV reactivation is associated with increased risk of death or whether it is simply another marker of critically-ill status including impairment of cell-mediated immunity is still controversial [6, 8, 14]. Because the meaning of CMV reactivation is unknown, treatment with patients with CMV reactivation has not been determined. Therefore, there are is no clinical guideline on when to administer antiviral agent for CMV reactivation in non-immunocompromised patients. This study could be one of the evidence suggesting that treatment of CMV reactivation in non-immunocompromised patients may not be necessary.

Patients who died within 30 days after CMV reactivation were excluded from the study because patients with severe state are more likely to receive ganciclovir and could show higher mortality rate. Despite excluding patients who died within 30 days after CMV reactivation, patients who stayed in ICU or hospitalized more than 30 days definitely received more ganciclovir treatment. CMV PCR titer was significantly higher in the ganciclovir treated group. Even if there is insufficient evidence of ganciclovir treatment in non-immunocompromised patients, it would be the result reflecting the efforts of the physicians to try anything to critically ill patients. However, there was no statistically significant association between ganciclovir treatment and 30-months survival in the multivariate analysis. This result was the same in the comparison between the CMV reactivation and inactivation groups. The result of this study that qSOFA scores correlate with CMV reactivation is the same result as studies confirming CMV reactivation found in critically-ill patients [7, 8, 19]. CMV as a potential risk factor for the development of cardiovascular disease has been the suggested by means of several epidemiologic, clinical, and laboratory studies [13, 21]. Therefore, we also assessed the development of cardiovascular events according to ganciclovir treatment in study patients and there was no statistically significance.

In the present study, we included patients with chronic obstructive pulmonary disease, interstitial lung disease, or connective tissue diseases. As a result, long-term or high-dose steroid users were also included. Strictly speaking, it could be difficult to say them non-immunocompromised. It was thought that patients with these underlying diseases were equally distributed in the ganciclovir treated group and non- ganciclovir treated group, so it would not have a significant effect on the study results.

Important questions would be remained whether such “non-immunocompromised” patients with severe illness still have CMV specific cell mediated immunity (CMI) enabling self-clearance of CMV replication. It is still unknown that the reactivation of CMV would be associated with the functionally of CMI in non-immunocompromised patients. The T-cell based immunodiagnostic assays of CMV (IGRA-CMV) have recently used to the risk stratification and diagnosis of CMV infection in the specific immunocompromised group. The IGRA-CMV has shown promising results in various immunocompromised patients such as SOT recipient or HSCT recipient [22]. However, the application of IGRA-CMV in clinical practice would be complicated because the optimal cut off values and ideal timing for IGRA-CMV were not known [23]. As most studies about IGRA-CMV are being conducted on high-risk patients of CMV infection, prospective studies on non-immunocompromised patients are likely to be needed in the future.

Our study has several limitations. First, it was a retrospective study, it is subject to confounding by indication. Treatment of ganciclovir were not randomized and was likely to be provided to patients with higher severity and higher risk of mortality. Systematic monitoring of CMV PCR titer was not performed and nearly half of patients had lost follow up within 30 months after discharge. Second, there is a potential selection bias because the test for CMV PCR was performed in a selective group of patients. Third, this study was based on patients at a single center and the results may not be generalizable to other population. Fourth, the side effects of drug in ganciclovir treated group could not be assessed. Finally, we do not know the serologic status of included patients. The prevalence of CMV-specific antibody varies in the worldwide, and seroprevalence in the Korean population has been reported to be relatively high. Although there are no nation-wide surveillance data in Korea, some studies targeted special group such as pregnancy and solid organ transplantation have reported 96–98% CMV IgG seroprevalence. Because we could assume that most patients in this study were CMV seropositive, we did not collect data on CMV-specific antibody results [12, 24].

In conclusion, our study showed that ganciclovir treatment was not associated with long-term prognosis. Antiviral treatment in this condition would not be necessary unless organ involvement is suspected.

## Supplementary Information

**Additional file 1: Table S1.** Association between baseline characteristics of patients and one-year survival. **Table S2.** Association between baseline characteristics of patients and 90-days survival. **Table S3.** Baseline characteristics of patients between CMV PCR-positive group and CMV PCR-negative group. **Table S4.** Association between baseline characteristics of patients and 30-months survival among CMV PCR-positive and negative patients.

## Data Availability

The datasets analyzed during the current study are available from the corresponding author on reasonable request.

## Abbreviations

CMV: Cytomegalovirus
HIV: human immunodeficiency virus
SOT: solid organ transplantation
HSCT: hematopoietic stem cell transplantation
RT-PCR: Reverse-transcriptase polymerase chain reaction
ICU: intensive care unit
CWIs: Charlson’s weighted index of comorbidity
qSOFA: quick sequential organ failure assessment
