# Supplementary material for: Impact of antiviral treatment on long-term prognosis in non-immunocompromised patients with CMV reactivation
Source: BMC Infect Dis. 2021 May 4;21:414. doi: 10.1186/s12879-021-06098-4 (PMC8094573; doi:10.1186/s12879-021-06098-4)
Supplement: Supplementary file 1 — Additional file 1: Table S1. Association between baseline characteristics of patients and one-year survival. Table S2. Association between baseline characteristics of patients and 90-days survival. Table S3. Baseline characteristics of patients between CMV PCR-positive group and CMV PCR-negative group. Table S4. Association between baseline characteristics of patients and 30-months survival among CMV PCR-positive and negative patients. [file 12879_2021_6098_MOESM1_ESM.docx]

**Table S1. Association between baseline characteristics of patients and one-year survival**

| **Variables** | **Univariate** | |  | **Multivariate** | |
| --- | --- | --- | --- | --- | --- |
|  | HR (95% CI) | P value |  | HR (95% CI) | P value |
| Sex | 1.389 (0.830-2.325) | 0.211 |  |  |  |
| Age | 0.992 (0.997-1.008) | 0.318 |  | 0.995 (0.978-1.012) | 0.543 |
| Ganciclovir treatment | 1.212 (0.752-1.955) | 0.430 |  | 1.533 (0.896-2.624) | 0.119 |
| Pneumonia | 1.959 (0.713-5.385) | 0.192 |  |  |  |
| ICU | 0.842 (0.503-1.409) | 0.513 |  | 0.513 (0.262-1.004) | 0.051 |
| Mechanical ventilation | 1.270 (0.705-2.287) | 0.427 |  |  |  |
| Quick SOFA | 1.277 (0.900-1.813) | 0.171 |  | 1.595 (1.049-2.426) | 0.029 |
| CWI | 0.850 (0.662-1.091) | 0.203 |  | 0.740 (0.423-1.295) | 0.292 |
| Hospital days > 30 days | 0.923 (0.574-1.486) | 0.742 |  |  |  |
| CMV PCR > 50,000 copies/ml | 0.932 (0.560-1.549) | 0.785 |  | 0.740 (0.423-1.295) | 0.292 |
| Progress to CMV disease | 0.261 (0.064-1.068) | 0.062 |  |  |  |
| Cardiovascular event | 1.451 (0.455-4.620) | 0.529 |  |  |  |

Abbreviations: ICU, intensive care unit; SOFA, sequential organ failure assessment; CWI, Charlson’s weighted index of comorbidity; CMV, Cytomegalovirus; PCR, polymerase chain reaction

**Table S2. Association between baseline characteristics of patients and 90-days survival**

| **Variables** | **Univariate** | |  | **Multivariate** | |
| --- | --- | --- | --- | --- | --- |
|  | HR (95% CI) | P value |  | HR (95% CI) | P value |
| Sex | 1.537 (0.642-3.682) | 0.334 |  |  |  |
| Age | 1.024 (0.993-1.057) | 0.125 |  | 1.030 (0.994-1.067) | 0.108 |
| Ganciclovir treatment | 2.076 (0.917-4.700) | 0.080 |  | 2.006 (0.654-6.158) | 0.224 |
| Pneumonia | 2.258 (0.305-16.691) | 0.425 |  |  |  |
| ICU | 2.659 (1.206-5.861) | 0.015 |  | 2.051 (0.760-5.533) | 0.156 |
| Mechanical ventilation | 1.695 (0.708-4.060) | 0.237 |  |  |  |
| Quick SOFA | 1.832 (1.044-3.214) | 0.035 |  | 1.318 (0.665-2.613) | 0.429 |
| CWI | 1.182 (0.798-1.72) | 0.405 |  | 1.007 (0.650-1.561) | 0.975 |
| Hospital days > 30 days | 1.031 (0.468-2.271) | 0.940 |  |  |  |
| CMV PCR > 50,000 copies/ml | 2.064 (0.942-4.525) | 0.070 |  | 2.291 (0.975-5.383) | 0.057 |
| Progress to CMV disease | 1.864 (0.558-6.229) | 0.312 |  |  |  |
| Cardiovascular event | 0.854 (0.115-6.310) | 0.877 |  |  |  |

Abbreviations: ICU, intensive care unit; SOFA, sequential organ failure assessment; CWI, Charlson’s weighted index of comorbidity; CMV, Cytomegalovirus; PCR, polymerase chain reaction

**Table S3. Baseline characteristics of patients between CMV PCR-positive group and CMV PCR-negative group**

| **Variables** | **CMV PCR-positive**  (n = 136) | **CMV PCR-negative**  (n = 131) | ***P* value** |
| --- | --- | --- | --- |
| **Sex , male** | 87 (64) | 82 (62.6) | 0.816 |
| **Age (years)** | 70 (60.3–78.8) | 70 (60.0–79.0) | 0.989 |
| **Pneumonia** | 124 (91.2) | 114 (87.0) | 0.276 |
| **Severity variables** |  |  |  |
| ICU | 46 (33.8) | 30 (22.9) | 0.048 |
| Mechanical ventilation | 26 (19.1) | 12 (9.2) | 0.020 |
| Quick SOFA | 1 (1.0–1.0) | 1 (0.0–1.0) | 0.005 |
| CWI | 0 (0.0–2.0) | 1 (0.0–2.0) | 0.462 |
| HD > 30 days after CMV reactivation | 73 (53.7) | 66 (50.4) | 0.590 |
| CMV PCR > 5,000 copies/ml |  |  |  |
| CMV DNAemia duration (days) | 35/104 (33.7) | 82/124 (66.1) | <0.001 |
| Progress to CMV pneumonitis | 55/123 (44.7) | 92/124 (74.2) | <0.001 |
| **Endpoint** | 107/132 (81.1) | 126/131 (96.2) | <0.001 |
| 30-months survival | 6 (4.4) | 4 (3.1) | 0.559 |
| One-year survival | 87 (64) | 82 (62.6) | 0.816 |
| 90-days survival | 70 (60.3–78.8) | 70 (60.0–79.0) | 0.989 |
| Cardiovascular events | 124 (91.2) | 114 (87) | 0.276 |

Data are expressed as number (%) of patients or median (IQR)

Abbreviations: ICU, intensive care unit; SOFA, sequential organ failure assessment; CWI, Charlson’s weighted index of comorbidity; HD, hospital day; CMV, Cytomegalovirus; PCR, polymerase chain reaction

**Table S4. Association between baseline characteristics of patients and 30-months survival among CMV PCR-positive and negative patients**

| **Variables** | **Univariate** | |  | **Multivariate** | |
| --- | --- | --- | --- | --- | --- |
|  | HR (95% CI) | P value |  | HR (95% CI) | P value |
| Sex | 1.020 (0.708-1.470) | 0.914 |  |  |  |
| Age | 0.998 (0.986-1.010) | 0.998 |  |  |  |
| CMV positivity | 0.896 (0.603-1.331) | 0.586 |  | 0.902 (0.606-1.344) | 0.613 |
| Pneumonia | 1.125 (0.681-1.860) | 0.645 |  |  |  |
| ICU | 1.045 (0.696-1.568) | 0.833 |  | 0.900 (0.577-1.403) | 0.641 |
| Mechanical ventilation | 1.006 (0.554-1.829) | 0.984 |  |  |  |
| Quick SOFA | 1.223 (0.966-1.550) | 0.095 |  | 1.253 (0.970-1.618) | 0.084 |
| CWI | 0.976 (0.843-1.131) | 0.748 |  | 0.977 (0.841-1.135) | 0.761 |
| Hospital days > 30 days | 1.100 (0.765-1.581) | 0.607 |  |  |  |
| CMV PCR > 50,000 copies/ml |  |  |  |  |  |
| Progress to CMV disease | 0.936 (0.297-2.944) | 0.909 |  |  |  |
| Cardiovascular event | 1.020 (0.708-1.470) | 0.914 |  |  |  |

Abbreviations: ICU, intensive care unit; SOFA, sequential organ failure assessment; CWI, Charlson’s weighted index of comorbidity; CMV, Cytomegalovirus; PCR, polymerase chain reaction
